# The complete chloroplast genomes of the mangrove fern *Acrostichum aureum*

**DOI:** 10.1080/23802359.2020.1772140

**Published:** 2020-06-01

**Authors:** Yan Zhong, Runxian Yu, Yongmei Chen, Renchao Zhou, Ying Liu

**Affiliations:** a State Key Laboratory of Biocontrol and Guangdong Provincial Key Laboratory of Plant Resources, School of Life Sciences, Sun Yat-sen University, Guangzhou, China; b School of Chemical Engineering, Sichuan University of Science & Engineering, Zigong, China

**Keywords:** *Acrostichum*, chloroplast genome, illumina sequencing, phylogenetic analysis

## Abstract

The mangrove fern *Acrostichum aureum* is widely distributed in the Indo West-Pacific and Atlantic East-Pacific regions. Here we assembled and annotated its chloroplast genome based on the Illumina sequencing reads. The complete chloroplast genome of *A. aureum* was 154,805 bp in length with the GC content of 38.38%. It contains a large single copy (LSC) region of 82,826 bp and a small single copy (SSC) region of 21,617 bp, separated by a pair of inverted repeat region (IRs) of 25,181 bp each. It contains 84 protein coding genes, 27 tRNA genes, and four rRNA genes. Phylogenetic analysis shows that *A. aureum* is closest to *Ceratopteris cornuta* in the subfamily Parkerioideae. The chloroplast genome of *A. aureum* reported here offers a useful resource for its phylogeography and conservation genetics studies.

*Acrostichum aureum* L. (Pteridaceae), also called mangrove fern, is the most widespread mangrove species distributed in both the Indo West-Pacific and Atlantic East-Pacific regions (Aksornkoae et al. 1992). How this species can disperse so widely remains unclear. Molecular markers can be used to assess its genetic diversity and phylogeographic pattern, which are useful to help understand its dispersal. So far, genomic resources for *A. aureum* have been very limited and only transcriptome data have been available (Zhang et al. 2016). Sequencing the chloroplast genome of *A. aureum* can provide useful genomic markers for assessing its genetic diversity and differentiation across its whole range in the world. This kind of genetic information is also essential for its conservation management. In this study, the complete chloroplast genome of *A. aureum* was sequenced using an Illumina sequencing platform. The assembled and annotated chloroplast genome sequence has been deposited in GenBank with the accession number MT379660.

DNA was extracted from fresh frond tissues of an individual of *A. aureum* sampled from Qinglangang, Wenchang, Hainan, China (110°47′31″E, 19°37′38″N). The voucher specimen (Yu20190815) was deposited in the herbarium of Sun Yat-sen University (SYS). A DNA library with an insert size of 350 bp was constructed and then sequenced on a HiSeq X Ten platform. A total of 6.0 Gbp paired end sequence data (PE = 150 bp) were used to assemble the chloroplast genome in NOVOPlasty (Dierckxsens et al. 2017) with the parameter kmer = 51 and the *rbcL* gene of *A. aureum* (GenBank accession number AB574794) as the seed. The assembled chloroplast genome was annotated using PGA (Qu et al. 2019) with default options and checked manually. The circular map of the chloroplast genome of *A. aureum* was generated using OrganellarGenomeDRAW (Lohse et al. 2013).

The complete chloroplast genome of *A. aureum* was 154,805 bp in length with the GC content of 38.38%. It contains a large single copy (LSC) region of 82,826 bp and a small single copy (SSC) region of 21,617 bp, separated by a pair of inverted repeat region (IRs) of 25,181 bp each. It was predicted to contain a total of 115 genes, including 84 protein coding genes, 27 tRNA genes, and four rRNA genes.

For phylogenetic analysis, the chloroplast genomes of 10 other species covering all five subfamilies of Pteridaceae were downloaded from GenBank. *Pecluma dulcis*, from the family Polypodiaceae was used as an outgroup. The sequences of 77 common genes of these species were concatenated and then aligned using MAFFT (Katoh and Standley 2013). A maximum likelihood phylogenetic tree was constructed with RAxML (Stamatakis 2014). As shown in the phylogenetic tree (Figure 1), *A. aureum* is closest to *Ceratopteris cornuta* in the subfamily Parkerioideae with 100% bootstrap support. The chloroplast genome of *A. aureum* reported here offers a useful resource for its phylogeography and conservation genetics studies.

**Figure 1.**
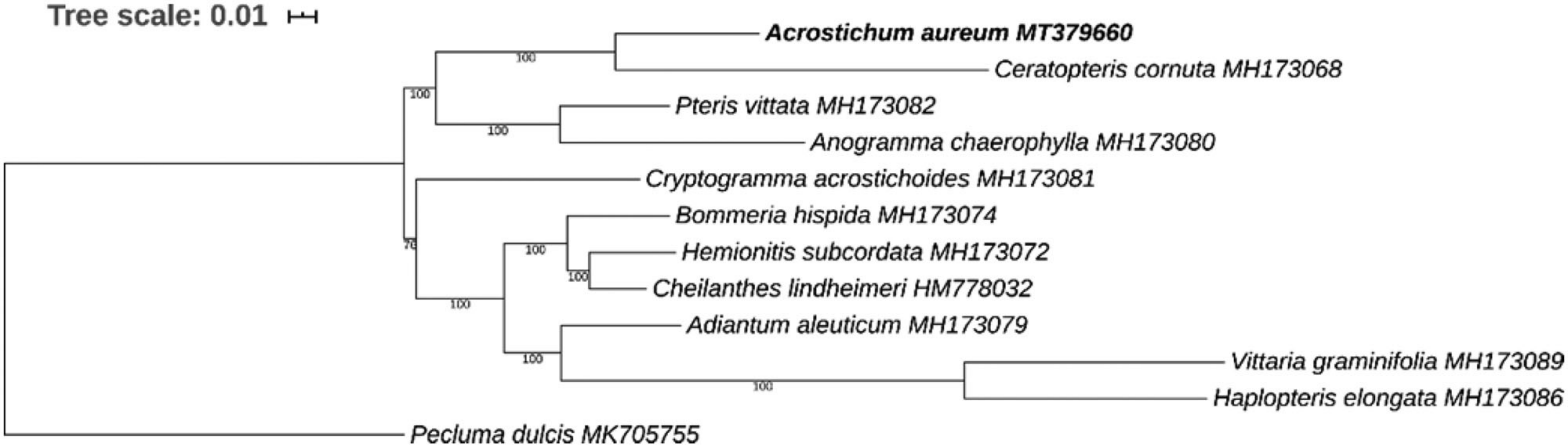
Phylogenetic analysis showing the position of *Acrostichum aureum* based on the concatenated sequences of 77 common chloroplast genes, with *Pecluma dulcis* as the outgroup. The phylogenetic tree was constructed by RAxML with bootstrap values on each node. The GenBank accession number was also shown for the chloroplast genome of each species.

## Data Availability

The chloroplast genome of the *A. aureum* was submitted to Genbank (https://www.ncbi.nlm.nih.gov/) under accession number: MT379660.
